# Supplementary material for: MetaRibo-Seq measures translation in microbiomes
Source: Nat Commun. 2020 Jun 29;11:3268. doi: 10.1038/s41467-020-17081-z (PMC7324362; doi:10.1038/s41467-020-17081-z)
Supplement: Supplementary file 10 — Supplementary Data 7 [file 41467_2020_17081_MOESM10_ESM.zip › File2/Confidence_High_Taxonomy/157245_out.krona.html]

Javascript must be enabled to view this page.

members
magnitude
magnitudeUnassigned
count
unassigned
taxon
rank

157245\_out

4

2
4
superkingdom

1224
4
phylum

28211
class
4

204455
4
order

4
family
31989

265

SRS013687\_contig\_number\_31602SRS023914\_contig\_number\_26542SRS051031\_contig\_number\_33567SRS1041145\_contig\_number\_13970
4
genus
